# Intermittent Hypoxia Increased the Expression of DBH and PNMT in Neuroblastoma Cells via MicroRNA-375-Mediated Mechanism

**DOI:** 10.3390/ijms23115868

**Published:** 2022-05-24

**Authors:** Shin Takasawa, Ryogo Shobatake, Yoshinori Takeda, Tomoko Uchiyama, Akiyo Yamauchi, Mai Makino, Sumiyo Sakuramoto-Tsuchida, Keito Asai, Hiroyo Ota, Asako Itaya-Hironaka

**Affiliations:** 1 Department of Biochemistry, Nara Medical University, 840 Shijo-cho, Kashihara 634-8521, Nara, Japan; rshobatake@naramed-u.ac.jp (R.S.); y-takeda@naramed-u.ac.jp (Y.T.); uchiyama0403@naramed-u.ac.jp (T.U.); yamauchi@naramed-u.ac.jp (A.Y.); m.makino@naramed-u.ac.jp (M.M.); ssumiyo@naramed-u.ac.jp (S.S.-T.); dc120002@naramed-u.ac.jp (K.A.); hiroyon@naramed-u.ac.jp (H.O.); iasako@naramed-u.ac.jp (A.I.-H.); 2 Department of Neurology, Nara Medical University, 840 Shijo-cho, Kashihara 634-8522, Nara, Japan; 3 Department of Obstetrics and Gynecology, Nara Medical University, 840 Shijo-cho, Kashihara 634-8522, Nara, Japan; 4 Department of Diagnostic Pathology, Nara Medical University, 840 Shijo-cho, Kashihara 634-8522, Nara, Japan; 5 Department of Respiratory Medicine, Nara Medical University, 840 Shijo-cho, Kashihara 634-8522, Nara, Japan

**Keywords:** intermittent hypoxia, sleep apnea syndrome, miR-375, neuroblastoma cells, hypertension, catecholamines, dopamine β-hydroxylase, phenylethanolamine *N*-methyltransferase

## Abstract

Sleep apnea syndrome (SAS), characterized by recurrent episodes of oxygen desaturation and reoxygenation (intermittent hypoxia (IH)), is a risk factor for hypertension and insulin resistance. We report a correlation between IH and insulin resistance/diabetes. However, the reason why hypertension is induced by IH is elusive. Here, we investigated the effect of IH on the expression of catecholamine-metabolizing enzymes using an in vitro IH system. Human and mouse neuroblastoma cells (NB-1 and Neuro-2a) were exposed to IH or normoxia for 24 h. Real-time RT-PCR revealed that IH significantly increased the mRNA levels of *dopamine β-hydroxylase* (*DBH*) and *phenylethanolamine N-methyltransferase* (*PNMT*) in both NB-1 and Neuro-2a. Western blot showed that the expression of DBH and PNMT in the NB-1 cells was significantly increased by IH. Reporter assays revealed that promoter activities of *DBH* and *PNMT* were not increased by IH. The miR-375 level of IH-treated cells was significantly decreased relative to that of normoxia-treated cells. The IH-induced up-regulation of DBH and PNMT was abolished by the introduction of the miR-375 mimic, but not by the control RNA. These results indicate that IH stress increases levels of DBH and PNMT via the inhibition of miR-375-mediated mRNA degradation, potentially playing a role in the emergence of hypertension in SAS patients.

## 1. Introduction

Sleep apnea syndrome (SAS) is a highly prevalent sleep disorder characterized by the repetitive partial or complete collapse of the pharynx during sleep. It is estimated that nearly one billion adults aged 30–69 in the world may suffer from SAS [1]. It induces apnea and hypopnea, which often result in a decreased oxygen saturation. A growing body of evidence suggests that SAS acts through recurrent episodes of oxygen desaturation and reoxygenation (intermittent hypoxia (IH)), which cause hypertension [2]. The pathophysiology of hypertension in cases of SAS is complex and dependent on various factors. The hypertension rate in SAS patients is two- to three-fold higher than that in the general population. Furthermore, habitual snoring, one of the hallmark symptoms of SAS, has been reported to be associated with hypertension in several epidemiologic studies [3,4]. Since SAS and hypertension share certain risk factors, i.e., the male gender, obesity, middle age, and a sedentary lifestyle, questions have been raised as to how much of this association may be attributable to these confounding variables. For the control of blood pressure, the sympathetic nervous system (catecholamine system), renin–angiotensin–aldosterone system and sodium reabsorption, and the endothelial system are important factors. We investigated how IH causes hypertension and report that IH up-regulates *renin* mRNA in juxtaglomerular cells via the down-regulation of microRNA (miR)-203 [5].

In the present study, we used catecholamine-producing human and mouse neuroblastoma cells and an in vitro IH system to investigate the direct effect of IH, a hallmark of SAS. An in vitro IH system is a controlled gas delivery system that regulates the flow of nitrogen and oxygen to generate IH [6,7]. We investigated the direct effect of IH on the gene expression(s) of catecholamine-synthesizing enzymes in neuroblastoma cells, and found significant increases in the mRNA levels of *dopamine β-hydroxylase* (*DBH*) and *phenylethanolamine N-methyltransferase* (*PNMT*) in response to IH treatment via the down-regulation of microRNA (miR)-375.

## 2. Results

### 2.1. Gene Expression Levels of DBH and PNMT in Human and Mouse Neuroblastoma Cells Were Increased by IH

**Figure 1:**
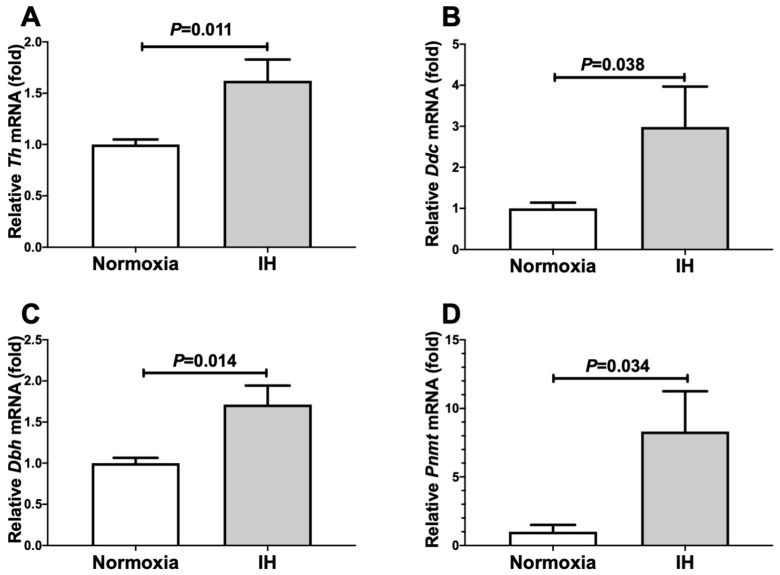
The mRNA levels of *Th* (**A**), *Ddc* (**B**), *Dbh* (**C**), and *Pnmt* (**D**) in mouse Neuro-2a cells subjected to normoxia or IH for 24 h. The levels of the catecholamine-synthesizing enzyme mRNAs were measured by means of a real-time RT-PCR using *rat insulinoma gene* (*Rig*)*/ribosomal protein S15* (*Rps15*) as an endogenous control. The data are expressed as the mean ± SE for each group of six independent experiments (n = 6). The statistical analyses were performed using Student’s *t*-test.

We exposed catecholamine-producing mouse Neuro-2a and human NB-1 neuroblastoma cells to normoxia or IH for 24 h. Following the IH treatment, we prepared cellular RNA and measured the mRNA levels of catecholamine biosynthesis enzymes such as *tyrosine hydroxylase* (*Th*), *L-3,4-dihydroxyphenylalanine* (*DOPA*) *decarboxylase* (*Ddc*), *dopamine β-hydroxylase* (*Dbh*), and *phenylethanolamine N-methyltransferase* (*Pnmt*) by means of a real-time reverse transcription-polymerase chain reaction (RT-PCR). We found that the mRNA levels of *Th*, *Ddc*, *Dbh*, and *Pnmt* were up-regulated by IH in mouse Neuro-2a cells (Figure 1).

**Figure 2:**
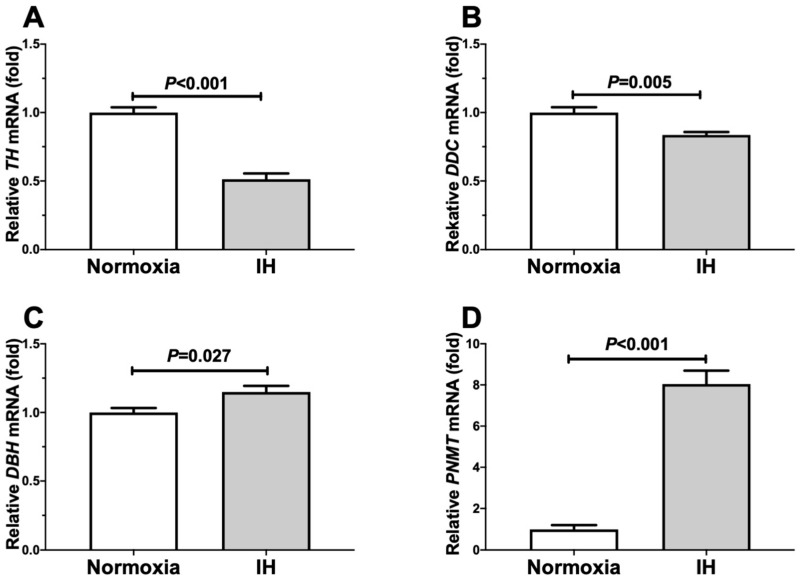
The mRNA levels of *TH* (**A**), *DDC* (**B**), *DBH* (**C**), and *PNMT* (**D**) in human NB-1 cells subjected to normoxia or IH for 24 h. The levels of the mRNAs were measured by means of a real-time RT-PCR using *β-actin* as an endogenous control. The data are expressed as the mean ± SE for each group of six independent experiments (n = 6). The statistical analyses were performed using Student’s *t*-test.

Following the treatment, we prepared cellular RNA and measured the mRNA levels of *TH*, *DDC*, *DBH*, and *PNMT* by means of a real-time RT-PCR. As shown in Figure 2, the mRNA levels of *DBH* and *PNMT* were significantly increased in the NB-1 cells in response to IH, although the *TH* and *DDC* mRNA levels did not increase. 

**Figure 3:**
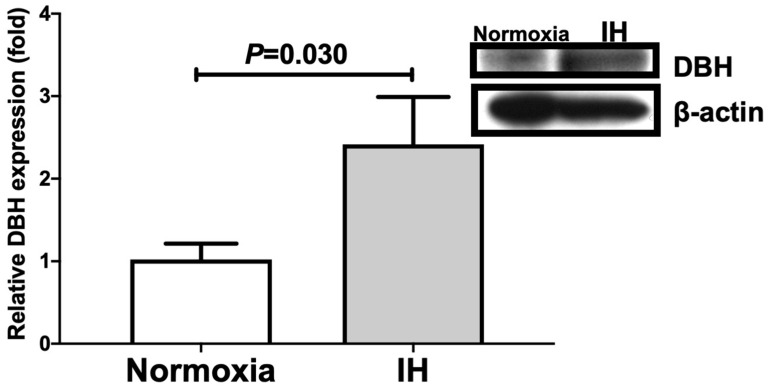
Relative protein expression of DBH in human NB-1 neuroblastoma cells subjected to IH. A representative immunoblot is shown in the right panel. The relative expression of DBH is arbitrarily presented. The DBH band densities were quantified using an image analysis and then normalized to β-actin, as measured in the same blot. Each bar represents the mean values of six independent experiments. The results are expressed as the mean ± SE in arbitrary units.

**Figure 4:**
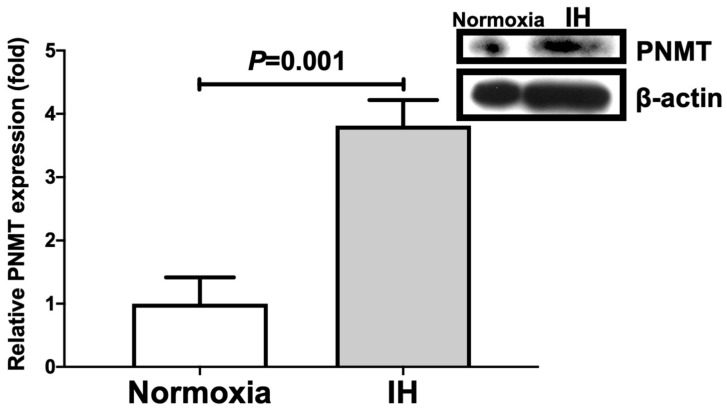
Relative protein expression of PNMT in human NB-1 neuroblastoma cells subjected to IH. A representative immunoblot is shown in the right panel. The relative expression of PNMT is arbitrarily presented. The PNMT band densities were quantified using an image analysis and then normalized to β-actin, as measured in the same blot. Each bar represents the mean values of six independent experiments. The results are expressed as the mean ± SE in arbitrary units.

We further measured cellular DBH and PNMT levels by means of immunoblot analyses. As shown in Figure 3 and Figure 4, IH significantly increased the cellular DBH and PNMT levels in NB-1 cells.

### 2.2. The Promoter Activities of DBH and PNMT Were Not Increased by IH

**Figure 5:**
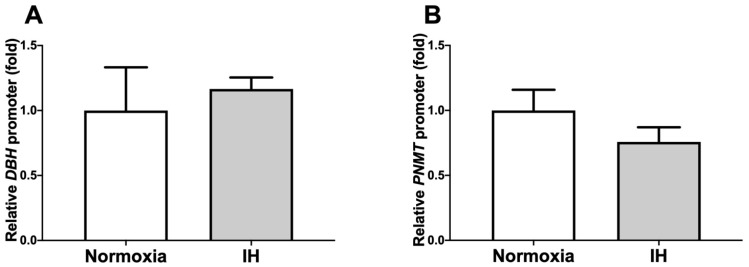
Promoter activities of *DBH* (**A**) and *PNMT* (**B**) in human NB-1 neuroblastoma cells. Reporter plasmids prepared by inserting the promoter fragments of *DBH* (−1018–+10) and *PNMT* (−600–+67) up-stream of a firefly luciferase reporter gene in a pGL4.17 vector were transfected into the NB-1 cells. After the cells were exposed to either IH or normoxia for 24 h, they were lysed, and the promoter activities of *DBH* and *PNMT* were measured. All data are presented as the mean ± SE of the samples of six independent experiments (n = 6). The statistical analyses were performed using Student’s *t*-test.

To determine whether the IH-induced increases in *DBH* and *PNMT* mRNAs were caused by the activation of transcription, a 1028 bp fragment containing 1018 bp of the human *DBH* promoter, and a 667 bp fragment containing 600 bp of the human *PNMT* promoter, were fused to the luciferase gene of pGL4.17 and transfected into NB-1 cells. After IH stimulation, we measured promoter activities and found that *DBH* and *PNMT* promoter activities were not increased by IH in NB-1 cells (Figure 5: *p* = 0.694 in *DBH* promoter and *p* = 0.242 in *PNMT* promoter). These results suggested that the gene expression of *DBH* and *PNMT* in response to IH was not regulated by transcription. 

### 2.3. The MiR-375 Level Was Significantly Decreased by IH

**Figure 6:**
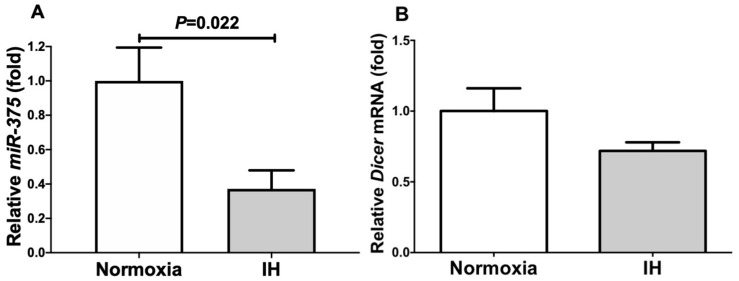
The levels of *miR-375* (**A**) and *Dicer* mRNA (**B**) in human NB-1 cells subjected to normoxia or IH for 24 h. The levels of *miR-375* and *Dicer* mRNA were measured by means of a real-time RT-PCR using *U6* (for the miR-375) and *β-actin* (for the Dicer) as endogenous controls. The data are expressed as the mean ± SE for each group of six independent experiments (n = 6). The statistical analyses were performed using Student’s *t*-test.

We considered a possible explanation that the IH-induced up-regulation of *DBH* and *PNMT* was controlled post-transcriptionally. Therefore, we searched for targeted miRNA using the MicroRNA.org program (http://www.microrna.org/microrna/home.do, 23 October 2021), which revealed that *DBH* and *PNMT* mRNAs had a potential target sequence for miR-375. There were no other miRNA candidates targeting both genes. We measured the miR-375 levels of IH-treated cells using real-time RT-PCR and found that the level was significantly lower than that of normoxia-treated cells (Figure 6: 0.371 ± 0.108 folds vs. normoxia, *p* = 0.022). There are several possible reasons as to why the level of miR-375 was decreased by IH; one is that the mRNA levels of some enzymes involved in miRNA biosynthesis/degradation are influenced by IH; another is that the level of miR-375 was specifically decreased by IH, either via decreased biosynthesis or enhanced degradation. We measured the *endoribonuclease Dicer* (*DICER*), which is involved in the biosynthesis of miRNAs [8,9], and found that its expression was unchanged by IH (Figure 6: *p* = 0.135). These results suggest that miR-375 plays a key role in the post-transcriptional regulation of the mRNA levels of *DBH* and *PNMT*. To investigate whether *DBH* and *PNMT* expression in IH is regulated by miR-375, miR-375 mimic and non-specific control RNA (miR-375 mimic NC) were introduced into NB-1 cells with IH/normoxia exposure, and the mRNA levels of *DBH* and *PNMT* were measured using real-time RT-PCR.

**Figure 7:**
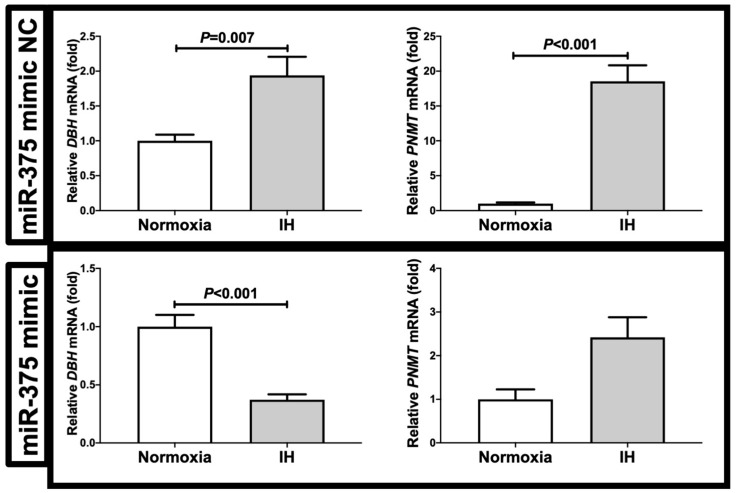
Effects of the miR-375 mimic transfection on *DBH* and *PNMT* expression. The miR-375 mimic and the non-specific control RNA (miR-375 mimic NC) were introduced into the NB-1 cells using Lipofectamine^®^ RNAiMAX just before the IH/normoxia exposure, and the mRNA levels of *DBH* and *PNMT* were measured by means of a real-time RT-PCR, as described in the “Materials and Methods” section, using *β-actin* as an endogenous control. The data are expressed as the mean ± SE for each group of six independent experiments (n = 6). The statistical analyses were performed using Student’s *t*-test.

As shown in Figure 7, we found that the IH-induced increases in *DBH* and *PNMT* mRNAs were abolished by the introduction of the miR-375 mimic, but not by the miR-375 mimic NC. These findings indicate that IH stress down-regulated the miR-375 level in human neuroblastoma cells (Figure 6) and that the levels of *DBH* and *PNMT* mRNAs were increased via the miR-375-mediated mechanism.

## 3. Discussion

SAS patients and their organs, tissues, and cells are exposed to IH. Thus, we exposed catecholamine-synthesizing neuroblastoma cells to IH in the present study and found that IH exposure induced increases in the *DBH* and *PNMT* mRNA levels in human neuroblastoma cells. We further studied the mechanisms by which IH up-regulates the mRNA levels of *DBH* and *PNMT* and found the possibility of post-transcriptional miRNA-regulated mechanisms. The expression of DBH and PNMT was up-regulated by IH via the miR-375-mediated mechanism. Hypertension in SAS patients could be caused by the IH-induced up-regulation of DBH and PNMT.

Recent epidemiological research demonstrated that SAS may be associated with various metabolic dysfunctions, including dyslipidemia, cardiovascular diseases, insulin resistance, and hypertension. The pathophysiology of hypertension in relation to SAS is dependent on various factors; for example, the sympathetic tone, peripheral vasoconstriction, altered baroreceptor reflexes, increased renin–angiotensin system activity [2,5], and increased plasma noradrenaline concentrations [10]. In particular, there are numerous reports concerning catecholamine synthesis in SAS patients and experimental models—more specifically, increased catecholamine secretion [11,12] the and up-regulation of the *PNMT* gene by IH [13]. Regarding catecholamine secretion from neuronal/adrenal chromaffin cells in hypoxia, hypoxia stimulates catecholamine secretion/gene expression in vitro and in vivo [14,15,16]. 

Previous studies focusing on catecholamine biosynthetic enzyme activities in the adrenal medulla, which is derived from the neural crest of spontaneously hypertensive rats (SHRs), showed inconsistent results. This was reported to be decreased [17,18,19], unchanged [20], and increased [21,22] in young SHRs. Ddc, Dbh, and Pnmt activities were decreased [18,23] or increased [24,25] in young SHRs, but unchanged in adult SHRs [18,25]. Due to the apparent correlation of the catecholamine biosynthetic enzyme activities, some authors have suggested that these genes may be co-regulated by a single locus [26].

We also investigated the mechanisms by which IH up-regulates the mRNA levels of *DBH* and *PNMT*. We found that the promoter activities of the genes were not increased by IH, which suggested that the IH-induced up-regulation of the *DBH* and *PNMT* mRNAs is regulated during the post-transcriptional step. miRNAs are small non-coding RNAs (≈22 nucleotides in length) that modulate gene expression by either translational suppression or the degradation of the mRNA through binding to the 3′-untranslated regions of the target genes in a base-pairing manner [27]. They affect the stability of their target mRNAs, resulting in changes in the amount of target mRNA, which is one of the mechanisms associated with post-transcriptional regulation. To date, a number of studies concerning the role of miR-375 have been reported to be influenced in normal pancreatic genesis [28], the proinflammatory macrophage response [29], pituitary prolactin synthesis [30], neuroendocrine differentiation and tumorigenesis in lung carcinoid cells [31], inhibition in proliferation and invasion of nasopharyngeal carcinomas [32], and inhibition in the proliferation, migration, and invasion of esophageal squamous cell carcinomas [33]. Several such studies have indicated that miRNAs are involved in the regulation of many biological processes (migration, metastasis, cell proliferation, apoptosis, chemosensitivity, etc.) in these various types of cells.

A few studies have addressed the correlation between miRNAs and hypertension in patients with SAS. For example, three plasma miRNAs (miR-378a-3p, miR-100-5p, and miR-486-5p) have been found to predict blood pressure responses to the continuous positive airway pressure treatment in patients with resistant hypertension and SAS [34]. Several miRs with altered expression in catecholamine-producing pheochromocytomas and paragangliomas (such as miR-15, -16, -21-3p, -96, -101-, -133, -137, -139-3p, -183, -195, -210, -338-3p, -375, -382, -483-5p, -488, -508, -765, -855, and -1225-3p) were reported [35]. In particular, there are a few reports on the function of miR-375, and they suggested it to be involved in the regulation of cytokines, extracellular matrix–receptor interaction, focal adhesion, phosphatidylinositol-3 kinase-protein kinase B (Akt), amoebiasis, and protein-processing pathways [36]. Single nucleotide polymorphisms (rs11174811 and rs3803107) in miR-375 target sites of the 3′-untranslated region in the arginine vasopressin receptor 1a gene were reported to be associated with the risk of hypertension [37]. However, these studies did not indicate the involvement of miR-375 in SAS patients’ hypertension. In the present study, the decline in miR-375 with a target sequence in the *DBH* and *PNMT* mRNAs could have contributed to the worsening hypertension in the IH condition induced by the up-regulation of the *DBH* and *PNMT* mRNAs. Therefore, it is quite important and necessary to clarify the relationships between miR-375, catecholamine-metabolizing enzyme expression, and hypertension using experimental animal models and/or clinical samples in the future. Additionally, since miRNA is easily measured in a clinical laboratory using clinically obtained blood samples of patients, it is possible that the measurement of miR-375 is used for the prediction and diagnosis of hypertension in SAS and/or habitual snoring patients.

In this study, the gene expression of *DBH* and *PNMT* was increased via the down-regulation of the miR-375 level in the IH-treated neuroblastoma cells. It is suggested that, in SAS patients, the up-regulation of *DBH* and *PNMT* in neural cells in the adrenal medulla may induce hypertension, while miR-375 could play a crucial role in the regulation of such gene expressions.

## 4. Materials and Methods

### 4.1. Cell Culture

The mouse neuroblastoma Neuro-2a cells utilized were purchased from the Japanese Collection of Research Bioresources Cell Bank (Ibaraki, Japan). The Neuro-2a cells were grown in E-MEM medium (FUJIFILM Wako Pure Chemical Corporation, Osaka, Japan) containing 10% (*v/v*) fetal calf serum (FCS), 100 units/mL penicillin G (FUJIFILM Wako), and 100 µg/mL streptomycin (FUJIFILM Wako), and human neuroblastoma NB-1 cells were grown in RPMI1640 medium (Nacalai Tesque, Inc., Kyoto, Japan) containing 10% (*v/v*) FCS, 100 units/mL penicillin G (FUJIFILM Wako), and 100 µg/mL streptomycin (FUJIFILM Wako), as described in prior studies [38,39,40]. The cells were exposed to either normoxia (21% O_2_, 5% CO_2_, and balanced N_2_) or IH (70 cycles of 5 min sustained hypoxia (1% O_2_, 5% CO_2_, and balanced N_2_) and 10 min normoxia) in a custom-designed, computer-controlled incubation chamber attached to an external O_2_-CO_2_-N_2_ computer-driven controller (O_2_ programmable control, 9200EX, Wakenyaku Co., Ltd., Kyoto, Japan), as described in previous studies [5,40,41,42,43,44,45,46]. We used this in vitro model of IH, which resulted in fluctuations in the pressure of oxygen similar to the IH condition observed in patients with severe SAS, to repeatedly expose the cells to severe hypoxemia followed by mild hypoxemia or normoxia (i.e., IH) [47]. We previously reported that the magnitude of the IH expressed by SpO_2_ fluctuated between 75% and 98% and between 50% and 80% in patients with SAS [6,7,48], which was nearly equivalent to the medium condition in the present study.

### 4.2. RT-PCR

Total RNA was isolated using an RNeasy Plus Cell Mini Kit (Qiagen, Hilden, Germany) for Neuro-2a and NB-1 cells, and cDNA was synthesized from total RNA as a template using a High-Capacity cDNA Reverse Transcription kit (Applied Biosystems, Foster City, CA, USA), as previously described [5,40,41,42,43,44,45,46,49,50,51]. A real-time polymerase chain reaction (PCR) was performed using an SYBR^®^ Fast qPCR kit (KAPA Biosystems, Boston, MA) and a Thermal Cycler Dice Real Time [JP1] System (Takara Bio, Kusatsu, Japan). All the PCR primers were synthesized by Nihon Gene Research Laboratories, Inc. (NGRL; Sendai, Japan), and the primer sequences for each primer set are described in Table 1 and Table 2. PCR was performed in an initial step of 3 min at 95 °C, followed by 40 cycles of 3 s at 95 °C and 20 s at 60 °C for *β-actin*; 45 cycles of 10 s at 95 °C, 5 s at 60 °C, and 20 s at 72 °C for *Rig/RpS15*; and 45 cycles of 3 s at 95 °C and 20 s at 60 °C for *Th* (human and mouse), *Ddc* (human and mouse), *Dbh* (human and mouse), *Pnmt* (human and mouse), *endoribonuclease Dicer* (*DICER*), and *microRNA-375* (*miR-375*). The mRNA expression levels were normalized to the mRNA level of *Rig/RpS15* in mouse samples or *β-actin* in human samples, and the *miR-375* level was normalized to the *U6* RNA level.

### 4.3. Construction of Reporter Plasmid and Luciferase Assay

Reporter plasmids were prepared by inserting the promoter fragments of human *DBH* (−1018–+10) and *PNMT* (−600–+67) up-stream of a firefly luciferase reporter gene in the pGL4.17 vector (Promega, Madison, WI, USA). The reporter plasmids were transfected into human NB-1 neuroblastoma cells using Lipofectamine^®^ 3000 (Invitrogen, Waltham, MA, USA), as previously described [5,40,43,45,46,50], and the cells were exposed to either 70 cycles/24 h of IH or normoxia for 24 h. After the cells were exposed to IH, they were harvested, and cell extracts were prepared in extraction buffer (0.1 M potassium phosphate, pH 7.8/0.2% Triton X-100; Life Technologies, Carlsbad, CA, USA). To monitor the transfection efficiency, pCMV-SPORT-βgal plasmid (Life Technologies) was co-transfected in all experiments at a 1:10 dilution. Luciferase activity was measured using a PicaGene luciferase assay system (Toyo-ink, Tokyo, Japan), and was normalized by the β-galactosidase activity, as described previously [5,40,41,43,46,49,50].

### 4.4. Immunoblot Analysis

The immunoblot analysis was performed using an NB-1 cell extract (5 × 10^5^ cells), as described in previous studies [5,39,52,53,54], an anti-DBH or -PNMT rabbit polyclonal antibody (Proteintech, Rosemont, IL, USA) and an anti-β-actin monoclonal antibody (Sigma, St. Louis, MO, USA) raised against Ac-Asp-Asp-Asp-Ile-Ala-Ala-Leu-Val-Ile-Asp-Asn-Gly-Ser-Gly-Lys, a SNAP id^®^ 2.0 Protein Detection System (Merck Millipore, Burlington, MA, USA), and an ECL Select detection reagent (Cytiva, Marlborough, MA, USA). The band intensities were analyzed using ImageJ software (National Institute of Health, Bethesda, MD, USA), as previously described [5,53,55,56].

### 4.5. MiRNA Extraction, Reverse Transcription, and Real-Time Quantitative PCR

Total RNA, including miRNA, was isolated from NB-1 cells using the miRNeasy mini kit (Qiagen) according to the manufacturer’s instructions. An equal amount of DNase-treated RNA was Poly-A-tailed using a Mir-X^TM^ miRNA first-strand synthesis kit (Clontech Laboratories, Inc., Mountain View, CA, USA) according to the manufacturer’s protocol. The conditions for PCR were 95 °C for 10 s, followed by 45 cycles of amplification (95 °C, 5 s, 60 °C, 20 s). U6 small nuclear RNA was used as an endogenous control for miRNA, as previously described [5,43,45,50]. The primers are listed in Table 2.

### 4.6. MiR-375 Mimic Transfection

MiR-375 mimic (5′-UUUGUUCGUUCGGCUCGCGUGAtt-3′, 5′-UCACGCGAGCCGAACGAACAAAtt-3′) and non-specific control RNA (miR-375 mimic NC) (5′-UUUGUACUACACAAAAGUACUGtt-3′, 5′-CAGUACUUUUGUGUAGUACAAAtt-3′) were synthesized by NGRL and introduced into NB-1 cells using Lipofectamine^®^ RNAiMAX (Invitrogen) [5,43,45,46,50] just before IH/normoxia exposure, and the mRNA levels of *DBH* and *PNMT* were measured by real-time RT-PCR, as previously described in Section 4.2.

### 4.7. Data Analysis

The results are expressed as mean ± SE. Statistical significance was determined by Student’s *t*-test using GraphPad Prism software (GraphPad Software, La Jolla, CA, USA).

## Figures and Tables

**Table 1:** PCR primers (mouse) for the real-time RT-PCR.

Target mRNA	Primer Sequence
*Th* (NM_009377)	5′-GCCAAGGACAAGCTCAGGAAC-3′
	5′-ATCAATGGCCAGGGTGTACG-3′
*Ddc* (NM_001190448)	5′-CTGCAGGCTTACATCCGAA-3′
	5′-TTGATCTCTGAAGCAGCT-3′
*Dbh* (NM_138942)	5′-GACTCAACTACTGCCGGCACGT-3′
	5′-CTGGGTGCACTTGTCTGTGCAGT-3′
*Pnmt* (NM_008890)	5′-GTGAAGCGAGTCCTGCCTATC-3′
	5′-AAGATGCCTTTGACATCATCTACC-3′
*Rig/RpS15* (NM_009091)	5′-ACGGCAAGACCTTCAACCAG-3′
	5′-ATGGAGAACTCGCCCAGGTAG-3′

**Table 2:** PCR primers (human) for the real-time RT-PCR.

Target mRNA/MiR	Primer Sequence
*TH* (NM_199292)	5′-GGAGTTCGGGCTGTGTAAGCA-3′
	5′-GACTGGTACGTCTGGTCTTGGTAGG-3′
*DDC* (NM_001082971)	5′-GAACAGACTTAACGGGAGCCTTT-3′
	5′-AATGCCGGTAGTCAGTGATAAGC-3′
*DBH* (NM_000787)	5′-GTGCTACATTAAGGAGCTTCCAAAG-3’
	5′-GGCCTCATTGCCCTTGGT-3′
*PNMT* (NM_002686)	5′-CTGACTCGGCCCCGGGCCAG-3′
	5′-GGCCTCCCCAGCCAGGTACC-3′
Dicer (NM_177438)	5′-GAGCTGTCCTATCAGATCAGGG-3′
	5′-ACTTGTTGAGCAACCTGGTTT-3′
*β-actin* (NM_001101)	5′-GCGAGAAGATGACCCAGA-3′
	5′-CAGAGGCGTACAGGGATA-3′
miR-375 (NR_029867)	5′-AGCCGTTTGTTCGTTCGGCT-3′
	5′-GTGCAGGGTCCGAGGT-3′
U6 (NR_004394)	5′-CTCGCTTCGGCAGCACA-3′
	5′-AACGCTTCACGAATTTGCGT-3′

## Abbreviations

DBH	Dopamine β-hydroxylase
DDC	DOPA decarboxylase
DOPA	L-3,4-dihydroxyphenylalanine
Dicer	Endoribonuclease Dicer
FCS	Fetal calf serum
IH	Intermittent hypoxia
miRNA	MicroRNA
PNMT	Phenylethanolamine *N*-methyltransferase
Rig	Rat insulinoma gene
RpS15	Ribosomal protein S15
SAS	Sleep apnea syndrome
SHRs	Spontaneously hypertensive rats
TH	Tyrosine hydroxylase
